# Controversies of Treatment Modalities for Cerebral Venous Thrombosis

**DOI:** 10.4061/2010/956302

**Published:** 2010-12-19

**Authors:** Maria Khan, Ayeesha Kamran Kamal, Mohammad Wasay

**Affiliations:** Department of Medicine (Neurology), Aga Khan University, Stadium Road, Karachi 74800, Pakistan

## Abstract

Cerebral vein thrombosis has been well recognized for nearly two centuries. However, therapeutic options for the condition are limited due to lack of large randomized trials. The various modalities reportedly used include antiplatelets, anticoagulation, fibrinolysis, and mechanical thrombectomy. Of these, antiplatelets are the least studied, and there are only anecdotal reports of aspirin use. Anticoagulation is the most widely used and accepted modality with favorable outcomes documented in two randomized controlled trials. Various fibrinolytic agents have also been tried. Local infusions have shown more promise compared to systemic agents. Similarly, mechanical thrombectomy has been used to augment the effects of chemical thrombolysis. However, in the absence of randomized controlled trials; there is no concrete evidence of the safety and efficacy of either of these modalities. Limited study series disclosed that decompression surgery in malignant CVT can be life saving and provides good neurological outcome in some cases. *Conclusion*. Overall therapeutics for CVT need larger randomized controlled trials. Anticoagulaion with heparin is the only modality with a reasonable evidence to support its use in CVT. Endovascular thrombolysis and mechanical thrombectomy are reserved for selected cases who fail anticoagulation and decompression surgery for malignant CVT with impending herniation.

## 1. Introduction

Cerebral venous thrombosis (CVT) is a well-recognized condition presenting in multiple different forms. It was first described in 1825 [1] in a man who suffered from headaches, seizures, and delirium for six months. His autopsy revealed superior sagittal sinus thrombosis. Since that initial description nearly two centuries ago, several case reports and case series have come out, but the condition continues to be a diagnostic and therapeutic challenge.

The estimated incidence is about 2 to 4/million/year [2] and about 75% of these are reportedly women [3]. A recent study in children <18 years found a higher incidence of 6.7/million/year [4]. Also CVT seems to be a bigger problem in the Asian world compared to the west. A report from India by Panagariya et al. suggests that CVT accounts for up to 40% of strokes in women and 50% of all young strokes [5, 6].

Risk factors for CVT have been highlighted in several studies and case series. The largest trial to date was the International study on cerebral vein and dural sinus thrombosis (ISCVT) [7]. This was a prospective, observational study from 21 different countries in which 624 patients with CVT were followed up for 16 months. In this study, 43.6% patients had more than one risk factors. The commonest cause was genetic or acquired thrombophilia (34.1%), followed by use of oral contraceptives or hormonal replacement therapy (58.6% of female patients) and local or systemic infection in 12.3%. 

Another study carried out by Khealani et al. reported systemic and central nervous system infection as the commonest predisposing cause in patients from Pakistan and Middle East. These were followed by postpartum state, homocysteinemia, and genetic thrombophilia. The contribution of oral contraceptive pill use was much lower at 3% [8].

The etiologic factors are therefore diverse and may remain obscure in up to 40%, most of whom are believed to have a genetic predisposition [9]. Broadly speaking, the common settings for CVT are puerpurium, infectious and inflammatory disease, malignancy, and oral contraceptives use [10]. CVT presents most commonly with headaches, focal or generalized seizures, neurologic deficits, or coma [11–14].

The course of the illness in CVT varies with death and dependency rates ranging from 8 to 40% [7, 15–17]. The ISCVT has identified several predictors of poor outcome, most notable being older age ( >37 years), male gender, seizures at admission, rapid evolution of thrombosis, the presence of focal deficits, and CNS infection and cancer [7].

Therapeutic options for CVT have been explored in several studies. At present, anticoagulation is the mainstay of treatment. However, ~40% of patients with CVT have concomitant propensity for intracranial hemorrhage and hence the apprehension on the part of the clinicians. 

In this paper, we intend to review the evidence for and against the available therapeutic options for CVT.

## 2. General Measures

The most dreaded complications of CVT are intracranial hypertension and cerebral herniation. The raised intracranial pressure is secondary to obstructed venous drainage leading to cerebral edema and intracranial hemorrhage which is often accompanying this condition. A review paper on mechanisms of damage in CVT [18] highlights multiple factors such as increased pressure in the dural sinus as well as increased venous flow velocities, rate of occlusion of the sinus, and superimposed cytotoxic as well as vasogenic edema. The paper also suggests that the extent of damage depends on the rate at which occlusion occurs and collaterals are formed.

Hydration is of paramount importance when patients present with signs and symptoms suggestive of CVT. Dehydration will make the condition worse by making the hypercoagulability worse. 

Another important measure to prevent sudden elevations of intracranial pressure would be to give antiepileptic cover to these patients. Focal or generalized seizures are more frequent in CVT than in any other type of stroke and this includes status epilepticus. The ISCVT reported seizures in 39% of their patients. Again no consensus exists on use of antiepileptic agents in CVT, but the risk is higher for those who present initially with seizures and in those who have supratentorial brain lesions including hemorrhage and edema [19]. The risk of developing seizures after CVT diagnosis is very low in patients who do not have these risk factors. Therefore, for the high-risk group, we would recommend antiepileptic drug use. The guidelines for treatment of seizures and for status epilepticus are the same as the standard epilepsy guidelines. 

In this acute phase, medical management to decrease intracranial pressure (ICP) is recommended. This includes head elevation, hyperventilation to a target PaCO2 of 30–35 mmHg, and use of mannitol [20]. 

Glucocorticoids like dexamethasone are used by many for controlling ICP. There is, however, no randomized trial to prove their efficacy. A study [21] analyzed data from the ISCVT on the use of glucocorticoids and failed to find any benefit. Therefore, routine use of these agents cannot be recommended.

It is not known whether use of mannitol or hyperventilation improves outcomes, but it definitely buys time before a more definitive procedure for controlling ICP (like hematoma evacuation and decompressive craniectomy) can be undertaken. There are no randomized controlled trials to support their use, however. 

Isolated intracranial hypertension is also a well-recognized form of presentation in patients with CVT [22]. Given the difference in prognosis, its management also differs from simple management of intracranial hypertension, which includes measures to decrease ICP. For those with underlying venous thrombosis, in addition to these measures, anticoagulation is recommended although more aggressive measures like mechanical thrombectomy are seldom required.

## 3. Specific Measures

These would be tailored around the underlying cause of cortical venous thrombosis. The major causes include prothrombotic conditions, either genetic or acquired which are the commonest cause, oral contraceptives and other drugs that cause a hypercoagulable state, pregnancy and the puerperium, malignancy, infections including CNS infections, ear, sinus, mouth, face, and neck infections and even systemic infectious disease, head injury, and mechanical precipitants like lumbar puncture and neurosurgical procedures [7]. For genetic prothrombotic conditions, there is no specific therapy available. For acquired conditions, it is tailored accordingly. For drug-induced CVT, the drug has to be discontinued along with other measures that follow in this paper. Malignancy requires its own specific therapy besides treatment for CVT. 

Spread of infection from a contiguous site is a well-known cause of dural sinus thrombosis. Particularly important sources are mastoidits and other middle-ear infections as well as ethmoid and frontal sinusitis. For these infections, leading to septic dural sinus thrombosis, high-dose antibiotics are the mainstay of treatment. Additionally, local collections of pus at these sites may have to be drained. Anticoagulation is not well studied but has been used particularly in cavernous sinus thrombosis with good results [23, 24]. For lateral and superior sagittal sinus thrombosis, the data is even more sparse with mixed results [25, 26]. Therefore, till more evidence is available, the physician will have to balance the risk of benefit of anticoagulation with the risk of hemorrhage in septic dural sinus thrombosis.

Patients with nephrotic syndrome have a much higher incidence of arterial as well as venous thrombosis compared to the general population [27]. There are several underlying mechanisms that lead to this increased risk. The general therapeutic measures for treatment of CVT in patients with nephrotic syndrome do not differ from management of the condition due to other causes. However, the underlying disease condition leading to nephrotic syndrome has to be separately addressed. Those patients who achieve remission of nephrotic syndrome may discontinue anticoagulation after six months following remission if there is no other indication for anticoagulation.

## 4. Antiplatelets

There are only anecdotal reports of use of aspirin for cortical venous thrombosis. No randomized trial or even a case series exist on the use of antiplatelet agents.

## 5. Anticoagulation

In 1941, Lyons [28] for the first time described the successful use of heparin in cerebral venous thrombosis. He reported two cases of infective cavernous sinus thrombosis that greatly benefited from a combination of antibiotics and heparin.

In 1985, Bousser et al. [29] retrospectively reviewed 38 patients with angiographically proven cerebral venous thrombosis. Twenty three of these patients received heparin, and clinical improvement was reported in all with 19 making a complete recovery. No deaths occurred in the heparin arm, and based on these findings the authors concluded that heparin was both safe and efficacious in patients with CVT.

In 1991, the first randomized controlled trial [15] on anticoagulation in CVT was published. Twenty patients with aseptic CVT were randomized, 10 to a placebo arm and 10 to heparin arm. A CVT severity scale was used to monitor the clinical course. Patients in the heparin arm showed a clear improvement at day 3 (*P* < .05), and the difference remained significant after 8 days of treatment (*P* < .01). After 3 months, 8 of the heparin-treated patients had a complete clinical recovery, and 2 had slight residual neurological deficits. In the placebo group, only 1 patient had a complete recovery, 6 patients had neurological deficits, and 3 patients died (*P* less than  .01). Three patients in the treatment group and 2 in the control group had ICH when therapy was started. However, no new cases of ICH occurred after initiation of heparin. Three patients in the control group experienced a new or worsened hemorrhage.

In this same report [15], Einhaupl et al. described an additional retrospective study on the relation between heparin treatment and ICH in CVT patients. 43 patients with CVT and ICH were studied. 27 of these patients were treated with dose-adjusted intravenous heparin after the ICH. Of these 27 patients, 4 died (mortality 15%), and 14 patients completely recovered. Of the 13 patients that did not receive heparin after ICH, 9 died (mortality 69%) and only 3 patients completely recovered. The authors concluded that anticoagulation with dose-adjusted intravenous heparin is an effective treatment in patients with CVT and that ICH is not a contraindication to heparin treatment in these patients. The study was criticized for its small sample size, use of an outcome measure that was not previously validated, and a significant delay from symptom onset to the initiation of therapy.

The other randomized trial to improve on the results of the above trial came in 1999 [30]. This was a double-blind placebo-controlled multicenter trial. Thirty patients were randomized to subcutaneous nadroparin (180 antifactor Xa units/kg per 24 hours) and 29 to matching placebo for 3 weeks (double-blind part of trial), followed by 3 months of oral anticoagulants for patients allocated nadroparin (open part). Patients with cerebral hemorrhage caused by sinus thrombosis were also included. After 3 weeks, 6 of 30 patients (20%) in the nadroparin group and 7 of 29 patients (24%) in the placebo group had a poor outcome, defined as death or Barthel Index score of <15 (risk difference, −4%; 95% CI, −25 to 17%; NS). After 12 weeks, 4 of 30 patients (13%) in the nadroparin group and 6 of 29 (21%) in the placebo group had a poor outcome, defined as death or Oxford Handicap Score of ≥3 (risk difference, − 7%; 95% CI, − 26% to 12%; NS). There were no new symptomatic cerebral hemorrhages. One patient in the nadroparin group had a major gastrointestinal hemorrhage, and 1 patient in the placebo group died from clinically suspected pulmonary embolism. Authors concluded that patients with cerebral sinus thrombosis treated with anticoagulants (low-molecular-weight heparin followed by oral anticoagulation) had a favorable outcome more often than controls, but the difference was not statistically significant. Anticoagulation proved to be safe, even in patients with cerebral hemorrhage.

A Cochrane review [2] was published in 2005 using these two trials for meta-analysis, and this concluded that based upon the limited evidence available, anticoagulant treatment for cerebral sinus thrombosis appeared to be safe and was associated with a potentially important reduction in the risk of death or dependency which did not reach statistical significance.

The results from the ISCVT [7] also support the use of anticoagulation, as in a retrospective review done recently showed a nonsignificant but definite trend towards improvement with anticoagulation. 

No trials have compared unfractionated heparin with low-molecular-weight heparin.

The recently published EFNS guidelines [20] based on Cochrane review; MEDLINE search; Cochrane Central Register of Controlled Trials (CENTRAL) recommend that anticoagulation should be given to all patients with CVT who do not have contraindications for anticoagulation. The recommended duration of oral anticoagulation varies depending on the underlying etiology. For transient risk factors, the recommendation is for 3 months, for idiopathic variety and for mild thrombophilia, 6–12 months, and for those with recurrent episodes of CVT or severe thrombophilia, the therapy should continue indefinitely.

Most of the reviews suggest that anticoagulation is safe even in patients who have evidence of intracranial hemorrhage, be it intracranial or subarachnoid.

In one review published by Wasay and Kamal in 2008 [31] on anticoagulation, however, the authors were strongly of the opinion that in the absence of randomized trials and hence statistical significance, anticoagulation cannot be recommended across the board for all CVT patients. They have stressed on studies where patients have recanalized even in the absence of any treatment.

In the Paediatric population, uncertainty regarding the therapeutic options continues. In the International Paediatric Stroke study [32], 84 neonates were diagnosed with CVT from 10 countries. In these patients, there were significant differences regarding use of antithrombotics and their indications. A recent single center prospective study [33] evaluated the safety and efficacy of anticoagulation in neonates and children and concluded that in this age group also, AC is safe and nontreatment results in thrombus propagation.

Based on these trials and reviews, anticoagulation continues to be the mainstay of treatment for CVT.

## 6. Fibrinolytic Agents

Despite the use of anticoagulant therapy, some patients continue to worsen. For these patients, use of fibrinolytic agents, particularly locally, has been tried.

For many years, agents like urokinase and streptokinase were of interest for clot lysis. Initially, people tried systemic urokinase and reported mixed results [34, 35]. A series of 5 patients affected by aseptic dural sinus thrombosis were treated with a combination of heparin sodium and urokinase, and complete clinical recovery was reported [36]. Then in the late 80s, some investigators tried local instead of systemic urokinase. In 1988 to improve the safety profile, a patient with dural sinus thrombosis was successfully treated with local urokinase infusion continued for 8 hours [37]. The patient recovered with very minimal deficits despite a small temporal hemorrhage. 

Following this report, several case reports and series with this modality were published. Barnwell and colleagues [38] reported three patients who were treated with a transjugular direct infusion of urokinase. The period of infusion ranged from 4 to 10 days. Two patients had both angiographic and clinical improvement of signs and symptoms, whereas the third only showed angiographic improvement. Another series reported seven patients treated with direct infusion of urokinase into the thrombosed sinus. The duration of infusion was a mean of 163 hours. All had angiographic improvement, and six out of seven had clinical benefit also [39].

In 1995, Horowitz et al. [40] reported a case series of 12 patients with CVT treated with selective catheterization and urokinase infusion. 4 of these had hemorrhages on preinfusion scans. Despite this, there was no major therapeutic morbidity. Eleven out of the 12 patients had sinus patency restored, and 10 of these had excellent clinical outcome.

For superior sagittal sinus thrombosis, Wasay et al. [41] conducted a nonrandomized comparison of local urokinase infusion with systemic heparin anticoagulation. Forty patients were enrolled, 20 received local urokinase followed by systemic heparin, and 20 received only systemic heparin. Discharge neurological function was better in the thrombolysis group than in the heparin group (*P* = .019), but hemorrhagic complications were also more with the thrombolysis group (*P* = .49). The authors concluded that local urokinase may be superior to systemic heparin alone in the treatment of superior sagittal sinus thrombosis.

Other agents have also been tried for thrombolysis. In one patient, local thrombectomy followed by streptokinase infusion proved very beneficial [42]. In yet another series [43], 12 patients were treated with local recombinant tissue plasminogen activator along with systemic heparin. The authors concluded that this combination shows promise but should be reserved for those without obvious hemorrhage. The time taken to restore flow was faster than with urokinase. 

Subsequently, a systematic review was published in 2003 [44] that included cases of cerebral venous and dural sinus thrombosis treated with fibrinolytics. 72 studies were included and no randomized clinical trial was found. Urokinase was the thrombolytic most frequently administered (76%) and majority had it locally infused (88%). ICH was reported in 17% of the patients, and in 5% it caused clinical deterioration. The conclusion, however, was that although thrombolytics appeared to be safe, their efficacy cannot be assessed from the published literature. 

In 2004, Cochrane review [45] also failed to identify any randomized controlled trial on thrombolytic use in CVT and concluded that there is no concrete evidence of the safety and efficacy of thrombolytic therapy in dural sinus thrombosis.

More recently, some more reviews have come out on the use of local thrombolytics. A series of 168 patients of CVT [46] treated with individualized endovascular treatment was published in January 2009. These included direct thrombolysis via internal jugular vein, injection of urokinase via common carotid artery, and stent angioplasty in venous sinus. They reported favorable outcomes with these individualized procedures. Another Chinese study [47] reported five patients with deteriorating neurological condition who underwent endovascular thrombolysis. The recovery was excellent in four of the five patients; one, however, could not be saved despite a decompressive craniectomy.

In November 2009, another review of studies done on thrombolysis was published [48]. The authors concluded that although there was some evidence of beneficial effects of chemical thrombolysis from the reported literature, there is a need for prospective trials. Till then most institutions continue to combine direct thrombolysis with systemic anticoagulation. An even more recent experience with 19 patients from India [49] suggests that intrasinus thrombolysis is safe and effective in patients with CVT who fail to respond to the conventional anticoagulation.

Based on the available evidence and in the absence of any randomized controlled trial on fibrinolytic use in CVT, its safety and efficacy are still questionable.

## 7. Mechanical Thrombectomy

Mechanical thrombectomy has been used to augment the effects of chemical thrombolysis and sometimes to avoid fibrinolytics due to high risk of hemorrhage. Several methods have been tried for mechanical thrombectomy. These include balloon angioplasty, stenting, clot maceration, and rheolytic thrombectomy [50, 51]. 

In 2003, Soleau et al. [52] published a review of 31 patients retrospectively evaluated from one institution. Four treatment strategies were identified, and complications and clinical outcomes were assessed for each group. Chemical thrombolysis was very effective in restoring sinus patency; however, 30% experienced hemorrhagic complications. 60% in this group made good clinical recovery. Patients who underwent mechanical thrombectomies demonstrated low hemorrhagic complications, and 88% had good clinical recovery.

Kirsch et al. in 2007 [51] published a retrospective review of four patients with CVT treated with transfemoral intravenous rheolytic thrombectomy. All four had good restoration of blood flow, and in three it was completely normalized. In these three, there was a rapid clinical improvement also. The authors concluded that this was a safe and effective method for selected patients with dural venous sinus thrombosis.

There is only a single prospective review of endovascular treatment in CVT which was published in 2008 [53]. 20 patients were enrolled, 12 of those were comatose, and 14 had hemorrhagic infarcts before thrombolysis. Thrombolysis was done by introducing a catheter through the internal jugular vein into the superior sagittal sinus, most underwent thrombosuction with a rheolytic catheter, combined with thrombectomy with a Fogarty catheter, followed by urokinase infusion. 6 patients died, 5 of whom had large infarcts and impending herniation even prior to thrombolysis. The authors concluded that thrombolysis can be effective for severe sinus thrombosis, but patients may deteriorate because of increased cerebral hemorrhage. 

Therefore, endovascular therapy is a promising modality but lacks randomized controlled trials for its evaluation. However, the available evidence supports its use along with chemical thrombolysis as well as alone for treatment of selected cases of CVT.

## 8. Decompressive Surgery

This modality has limited utilization so far in the treatment of CVT. Decompression has been used in cases of malignant elevations in intracranial pressure to abort impending herniation. Despite treatment with endovascular thrombolytics, it was observed that there was poor outcome in patients with impending herniation. For these patients, decompressive craniectomy was introduced. Coutinho et al. [54] reports 3 such patients who underwent decompression, two had excellent outcome, the third died. 

Another prospective series [55] of three patients with malignant sinus thrombosis reported good recovery in two of these patients, and one was left with moderate disability. The authors reviewed prior literature on decompressive surgeries in these patients between 1970 and 2008. Eight such patients had been reported in three reviews [56–58], and the outcome in four was good. Three patients were left with moderate and one with severe disability. The authors concluded that decompressive surgery in these patients with severe CVT and evidence of malignant intracranial hypertension can be life saving, and the outcome can also be reasonably good. 

A retrospective review [59] of 12 patients with malignant cerebral venous thrombosis has recently been published. Of these, 8 underwent decompressive surgery. The four patients who did not undergo surgery died. Of these 8 who did, one died of pulmonary embolism, but the other 7 not just survived, but 6 of them made excellent neurological recovery.

Based on the current available literature, decompressive surgery can be a very promising option for patients with malignant cerebral venous thrombosis.

## 9. Conclusion

After reviewing all the available therapeutic modalities for CVT, the question remains which modality to use in which situation. Generally speaking, the choice of therapy, which is at the discretion of the treating physician in the absence of clear cut guidelines, depends largely on the clinical presentation. This may vary from mild headache to focal deficits and even coma. The presentation broadly speaking can be acute, subacute, or chronic. For subacute or chronic cases, headache is often the most prominent presenting feature, and the need is for urgent but not emergent therapy. Anticoagulation with heparin is the best studied and the only recommended therapy in these cases. Acute presentation can be more striking with encephalopathy and focal deficits dominating the picture. In these patients, again the best evidence is in favor of anticoagulation, but more aggressive measures to recannulate the sinus may be undertaken in case the patient continues to deteriorate. These include endovascular thrombolysis and mechanical thrombectomy.

Escalation of therapy is recommended in case of clinical worsening. A patient presenting with mild symptoms may just be managed on anticoagulation. However, if there is evidence of deterioration in terms of new deficits and drowsiness, therapy may be escalated to use of systemic or local thrombolytics, and in the presence of intracranial hemorrhage and malignant intracranial hypertension, surgical options may also be considered. 

To summarize, overall therapeutics for CVT need larger randomized controlled trials. Anticoagulation with heparin is the only modality with reasonable evidence to support its use in CVT, even in patients with cerebral hemorrhage. Endovascular thrombolysis is a promising option for patients with a severe form of CVT or following a failure of anticoagulation therapy. Mechanical thrombectomy is reserved for selected cases and decompression surgery for malignant CVT with impending herniation.
